# Subcategorization of suspicious non-mass-like enhancement lesions(BI-RADS-MRI Category4)

**DOI:** 10.1186/s12880-023-01144-w

**Published:** 2023-11-10

**Authors:** Dandan Liu, Zhaogui Ba, Yan Gao, Linhong Wang

**Affiliations:** Department of Radiology, The Eighth People’s Hospital of Jinan, No. 68, Xinxing Road, Gangcheng District of Jinan, Jinan, Shandong 271126 P. R. China

**Keywords:** Breast, Non-mass-like enhancement, Magnetic resonance imaging, Subcategorization, BI-RADS

## Abstract

**Background:**

This study aims to providing a reliable method that has good compliance and is easy to master to improve the accuracy of NMLE diagnosis.

**Methods:**

This study retrospectively analyzed 122 cases of breast non-mass-like enhancement (NMLE) lesions confirmed by postoperative histology. MRI features and clinical features of benign and malignant non-mass enhancement breast lesions were compared by using independent sample *t* test, *χ*^*2*^test and *Fisher* exact test. *P* < 0.05 was considered statistically significant. Statistically significant parameters were then included in logistic regression analysis to build a multiparameter differential diagnosis modelto subdivide the BI-RADS Category 4.

**Results:**

The distribution (odds ratio (OR) = 8.70), internal enhancement pattern (OR = 6.29), ADC value (OR = 5.56), and vascular sign (OR = 2.84) of the lesions were closely related to the benignity and malignancy of the lesions. These signs were used to build the MRI multiparameter model for differentiating benign and malignant non-mass enhancement breast lesions. ROC analysis revealed that its optimal diagnostic cut-off value was 5. The diagnostic specificity and sensitivity were 87.01% and 82.22%, respectively. Lesions with 1–6 points were considered BI-RADS category 4 lesions, and the positive predictive values of subtypes 4a, 4b, and 4c lesions were15.79%, 31.25%, and 77.78%, respectively.

**Conclusions:**

Comprehensively analyzing the features of MRI of non-mass enhancement breast lesions and building the multiparameter differential diagnosis model could improve the differential diagnostic performance of benign and malignant lesions.

## Background

Non-mass-like enhancement (NMLE) lesions are defined as areas of abnormal enhancement that do not have the characteristics of a mass and have no significant mass effect or well-defined borders [1]. These lesions are usually mixed with normal glands and adipose tissue. Because of the wide range of pathological types and the lack of typical imaging manifestations, NMLE has always been a difficult subject in breast magnetic resonance imaging (MRI) diagnosis and tends to be missed or misdiagnosed. Thus, the Breast Imaging Reporting and Database System (BI-RADS) classification relies more on the experience of diagnosticians. The diagnosis of NMLE is more difficult for younger physicians than for senior physicians. BI-RADS category 4 lesions have a wide range of malignant possibilities (2%-95%) [2, 3], and compared with mass-like enhancement lesions, the probability of diagnosing a lesion as category 4 is even higher,which will lead to an unnecessary fine needle aspiration biopsy for many benign lesions, and there are some false negative fine needle aspiration biopsies [4].

Some studies showed that the subcategorization of category4 is feasible [5–7], but the subclassification of BI-RADS category 4 lesions relied on the experience of radiologist [5, 7]. Almeida et al. [8]. used DCE-MRI for subcategorization and proposed that DWI could be used for subdivision of BI-RADS category 4 lesions, but did not give how to use ADC values. In our previous studies, Fischer’s scoring and ADC value were used to subdivide BI-RADS 4 lesions [9], but we found that the subcategorization of NMLE is not very accurate in the clinical practice. At present, there is no simple and quantifiable scoring scheme that can accurately subdivide BI-RADS category 4 breast lesions.

Therefore, the authors retrospectively investigated a group of NMLE lesions, analyzed their various MRI signs and established a multiparametric scoring model for the differentiation of benign and malignant NMLE. We also preliminarily explored the BI-RADS categorization and subcategorization of NMLE lesions, aiming at providing a reliable method that has good compliance and is easy to master to improve the accuracy of NMLE diagnosis by physicians.

## Methods

### General data

A total of 122 cases of breast NMLE lesions confirmed by postoperative histology were collected in our hospital from July 2013 to September 2022. Among the 45 cases of benign lesions, there were 7 cases of intraductal papilloma, 9 cases of abscess formation, 8 cases of chronic inflammation, one case of sclerosing adenosis, and one case of fat necrosis, and the rest were proliferative changes with or without fibromatous structure formation and ductal dilatation with periductitis. Among the 77 cases of malignant lesions, there were 29 cases of intraductal carcinoma in situ (5 cases with early invasion), 20 cases of invasive ductal carcinoma, 9 cases of invasive lobular carcinoma, 6 cases of invasive ductal-lobular carcinoma, 4 cases of invasive intraductal carcinoma, 3 case of intraductal papillary carcinoma, and one case of mucinous carcinoma. All of the cases had a single lesion. Inclusion criteria: 1. There was no treatment history before the MRI examination, and the surgical treatment was performed within 2 weeks after the MRI examination. 2. The imaging data were complete, and the dynamic contrast-enhanced scan showed NMLE. 3. The postoperative pathological data were complete. All patients were female, aged 29 to 78 years, with an average of 49.67 ± 11.12 years.

This study was approved by the Ethics Committee of The Eighth People’s Hospital of Jinan and all methods were also performed in accordance with the relevant guidelines and regulations under the committee supervision. Informed consent was obtained from these patients.

### Equipment and parameters

All patients underwent preoperative, multiphase dynamic noncontrast, contrast-enhanced, and diffusion-weighted imaging (DWI) MRI scans. A GE 1.5 T HDe superconducting MR imaging system and a 4-channel breast dedicated surface coil were used. Single-shot echo-planar imaging (EPI) for DWI examination was performed using the following settings: a repetition time (TR) = 8400 ms, an echo time (TE) = 93.8 ms, b-values = 0, 800 s/mm^2^, matrix = 128 × 128, and the number of excitations (NEX) = 2. The fat-suppressed T2-weighted imaging (T2WI) fast spin-echo (FSE) sequence was obtained using the following settings: chemical frequency-selective fat saturation, TR = 4660 ms, TE = 89.2 ms, matrix = 320 × 256, and NEX = 2. Both the DWI and T2WI sequences were transaxial scans with consistent positioning. The field of view was 320 mm × 320 mm, the slice thickness was 4 mm, and the interval was 1 mm, with 32 slices covering the entire breast. Dynamic contrast-enhanced scanning was performed using a VIBRANT sequence, with TR = 4.7 ms, TE = 2.2 ms, matrix = 320 mm × 320 mm, and slice thickness = 1.0 mm, covering the breast and axilla. A total of 12 scans were performed, with a single-scan time of 41 s, of which the first was a pre-scan, and at the end of the first scan, a bolus injection of the contrast agent gadolinium-diethylenetriamine pentaacetic acid (Gd-DTPA) at 0.1 mmol/kg was delivered via the cubital vein at a flow rate of 2 mL/s.

### Image observation and data measurement method

All scanned images were sent to the ADW4.3 workstation for post-processing. The morphological characteristics of the lesions were determined based on the dynamic contrast-enhanced images, and the distribution and internal enhancement characteristics of the lesions were observed and recorded according to the BI-RADS method (2013 edition). The T2WI signal characteristics of the lesions were recorded using the pectoralis major muscle as a reference. Time-intensity curve (TIC) measurement: The region of interest was placed in the area with the most significant enhancement, and the measurement was repeated three times. The curve that was most likely to indicate malignancy was selected as the TIC of the lesion, and its early enhancement rate at 1 min was calculated. Referring to the Fischer score an early enhancement rate of less than 50% was defined as grade 0, 50%-100% as grade 1, and > 100% as grade 2. Apparent diffusion coefficient (ADC) measurement: The region of interest was determined according to the images from dynamic contrast-enhanced examination. Three regions of interest were selected for the measurement, and the mean value was taken. Receiver operating characteristic (ROC) curves were used to determine the optimal diagnostic cut-off value for benign lesions, with those less than the optimal cut-off value considered to be malignant. Determination of vascular signs: The subtraction images with the most obvious enhancement were used for maximum intensity projection reconstruction to observe whether there were abnormally increased and thickened vessels in and around the lesion. Blood vessels with a length ≥ 3 cm and a maximum diameter ≥ 2 mm were used as the screening criteria [3], and a difference in blood vessel count ≥ 2 between the two breasts was considered an asymmetrical increase in breast blood supply; if one or more vessels entered the lesion, they were considered the feeding vessels. If one or both of the above criteria were satisfied, then the adjacent vascular sign was considered positive. Acquisition of the characteristics and parameters of all lesions was completed by two collaborating physicians with many years of work experience in breast MRI.

### Statistical methods

Medcalc12.7 statistical software was used for statistical analysis. The ROC curve was used to determine the optimal diagnostic cut-off value for ADC values. A chi-square test was used to analyze the differences in the distribution, internal enhancement characteristics, and T2WI signal characteristics of benign and malignant lesions. Logistic regression analysis was used to determine the multimodal scoring model, and the ROC curve was used to determine its diagnostic efficacy. *P* < 0.05 was considered statistically significant.

## Results

### Analysis of lesion morphology, T2WI signal, and other accompanying signs

Of the 122 lesions, there were 14 cases of linear distribution, 68 cases of segmental distribution (including distribution along the duct), 23 cases of local distribution, 11 cases of regional distribution, 4 cases of multiple regional distribution, and 2 cases of diffuse distribution. There were 6 cases of homogeneous enhancement, 55 cases of heterogeneous enhancement, 56 cases of clustered enhancement, and 9 cases of clustered ring enhancement. There were 3 cases of hypointense T2WI signal, 36 cases of isointense signal, 51 cases of slightly hyperintense signal, and 32 cases of hyperintense signal. There were 7 cases with ductal dilatation, 8 cases with adjacent skin thickening, and 5 cases with nipple retraction. See Table 1 for details.

**Table 1:** Value of each sign for the diagnosis of benign and malignant lesions

	Benign or malignant		Fisher’s exact test or *X*^2^ test	OR value
	Benign	Malignant		
**Distribution**				
Linear	7	7	*P* < 0.01	8.70 (*P* = 0.0001)
Segmental (including the distribution along the duct)	11	57		
Focal	14	9		
Regional	9	2		
Multiregional	4	0		
Diffuse	0	2		
**Enhancement pattern**				
Homogeneous	2	3	*P* < 0.01	6.29 (*P* = 0.0035)
Heterogeneous	29	26		
Clustered	6	46		
Clustered ring	7	2		
**T2WI signal**				
Hypointense	1	2	*P* = 0.559	
Isointense	11	25		
Slightly hyperintense	18	33		
Hyperintense	15	17		
**Enhancement rate**				
Less than 50%	3	0	*P* = 0.056	
50–100%	16	24		
Greater than 100%	26	53		
**TIC curve**				
Type I	20	6	*P* < 0.01	
Type II	24	56		
Type III	1	15		
**Vascular sign**				
Positive	10	54	*P* < 0.01	4.56 (*P* = 0.0072)
Negative	35	23		
**ADC value**				
> 1.40 × 10^–3^ mm^2^/s	23	12	*P* < 0.01	2.84 (*P* = 0.0008)
≤ 1.40 × 10^–3^ mm^2^/s	22	65		

### ADC value and early enhancement rate, enhancement curve, and vascular sign analysis

**Fig. 1:**
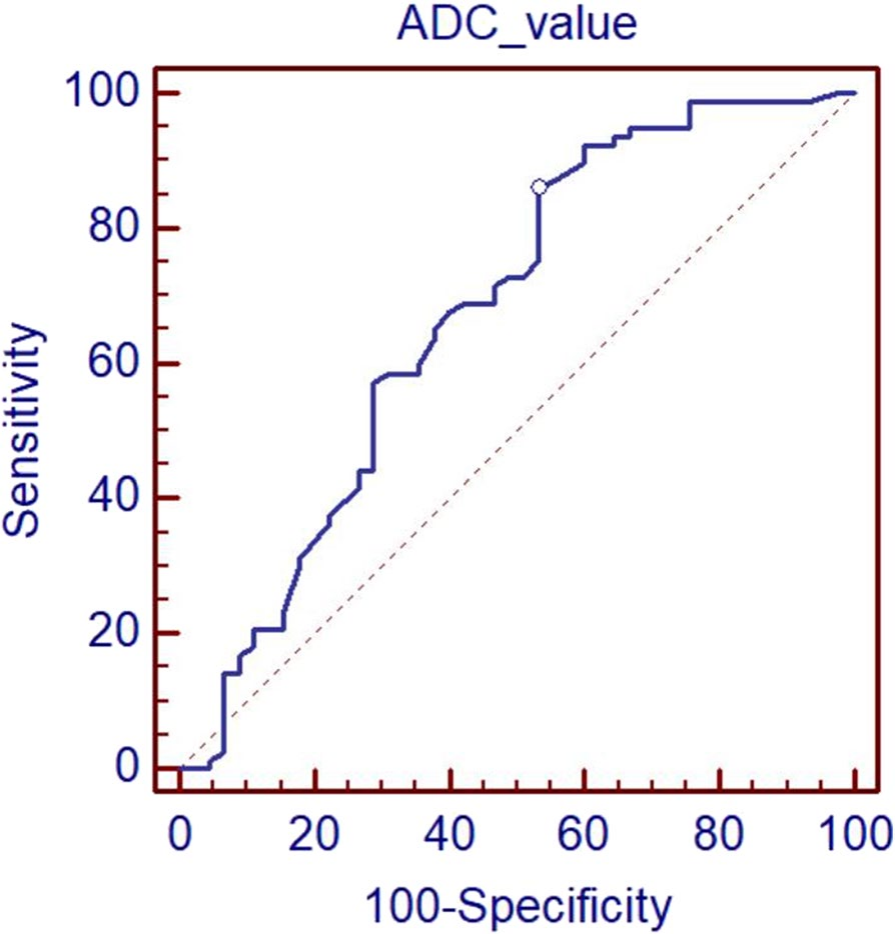
ROC curve showing the optimal cut-off value of the ADC value at 1.40 × 10^–3^ mm^2^/s. The area under the curve was 0.673 the sensitivity was 85.71%, and the specificity was 46.67%

The ADC values of the 81 lesions ranged from 0.63 to 2.03 × 10^−3^ mm^2^/s; with significantly higher values for benign lesions than for malignant lesions (1.37 ± 0.43 *VS* 1.14 ± 0.25, *t* = 3.82, *P* < 0.001); the optimal cut-off value of ROC analysis was 1.40 × 10^−3^ mm^2^/s, the area under the curve was 0.673, the sensitivity was 85.71 (95% confidence interval (CI): 75.9–92.6%), and the specificity was 46.67% (95% CI: 31.7–62.1%). See Fig. 1. The early enhancement rate ranged from 20 to 196%. In particular, the early enhancement rate was < 50% in 3 cases, 50 – 100% in 40 cases, and > 100% in 79 cases. There were 26 cases of inflow curve(type I), 80 cases of plateau curve (type II), and 16 cases of washout curve (type III). For details, see Table 1.

### Multimodal scoring

**Fig. 2:**
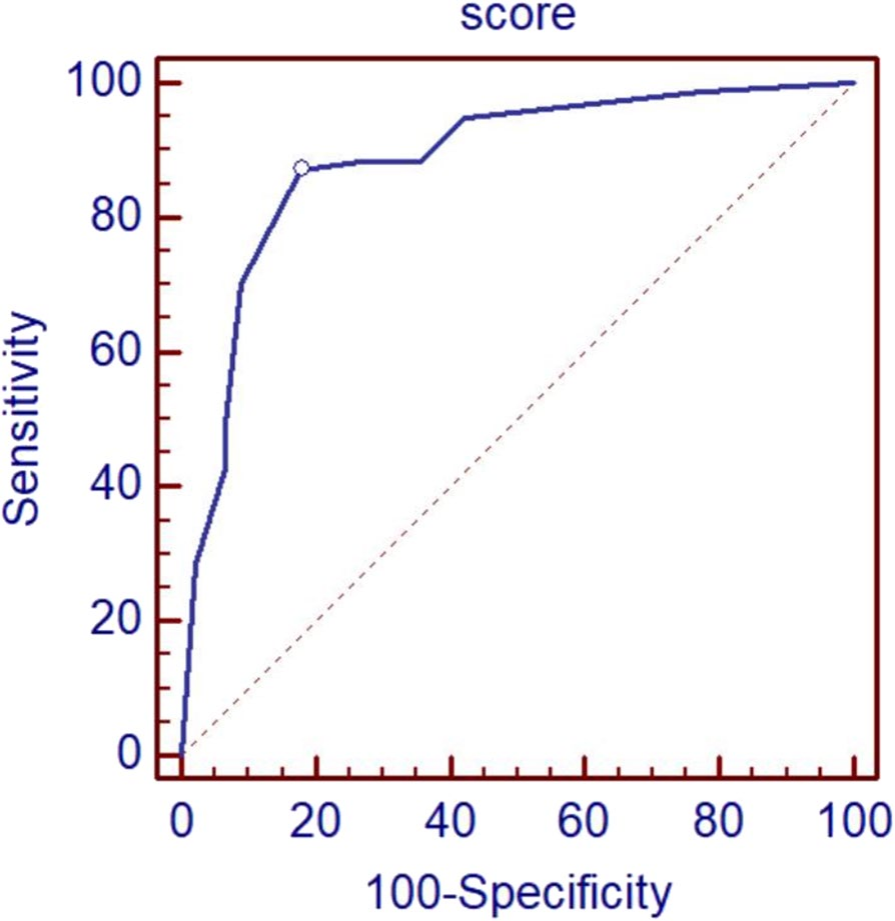
ROC analysis showing the optimal cut-off value of multimodal scoring at 4 points. The diagnostic specificity and sensitivity were 87.01% (95% CI: 77.4%-93.6%) and 82.22% (95% CI: 67.9%-92.0%), respectively

**Fig. 3:**
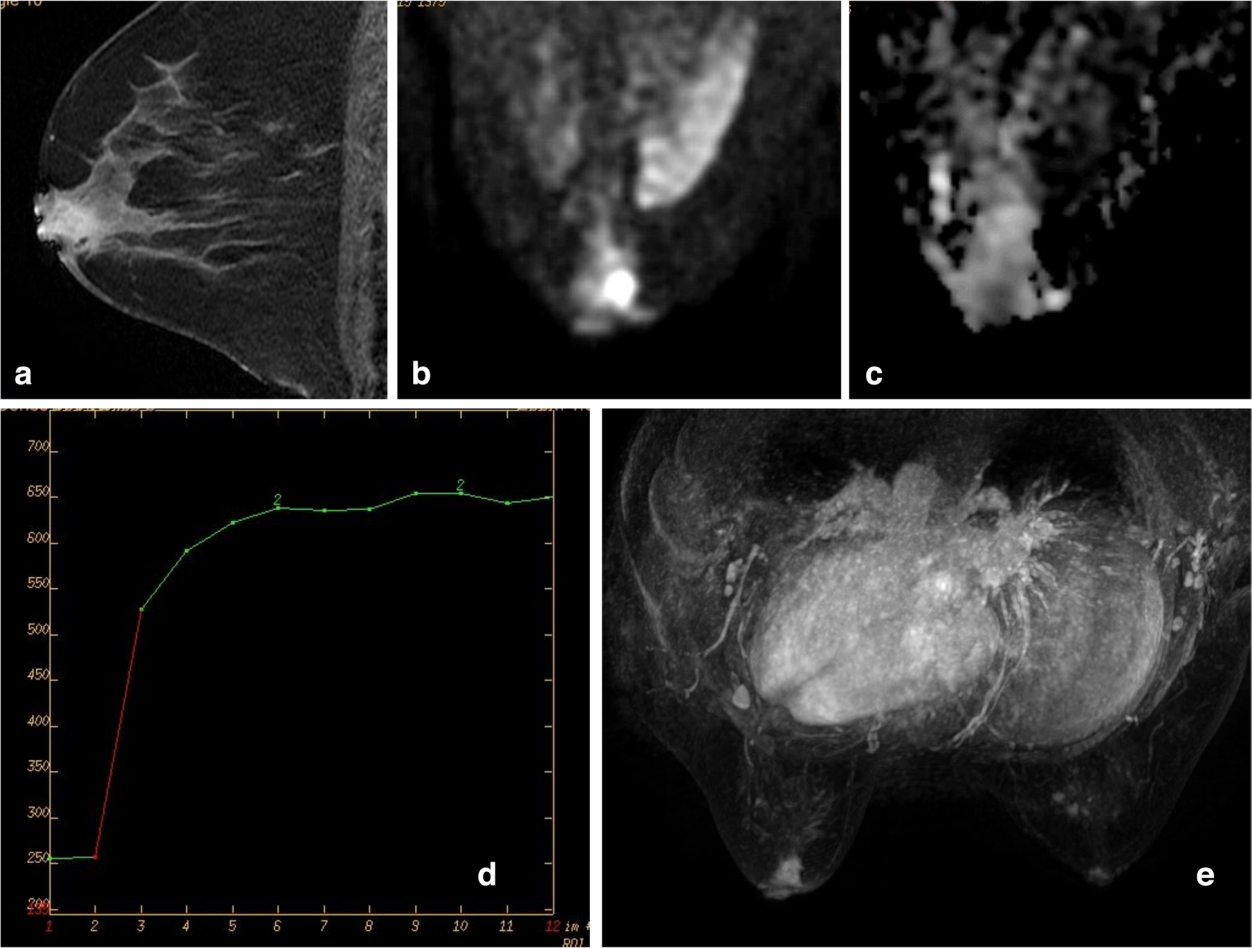
**a** A lesion in a 39-year-old female with focal and heterogeneous enhancement in theleft breast. **b** and **c** The ADC value was 0.93 × 10^–3^ mm^2^/s. **d** The imaging revealed a type TICII curve. **e** No increasing in vasculature. The multiparametric score was 2, the lesion was determined as BI-RADS-MRI type 4a. The pathological finding was inflammation with abscess formation

**Fig. 4:**
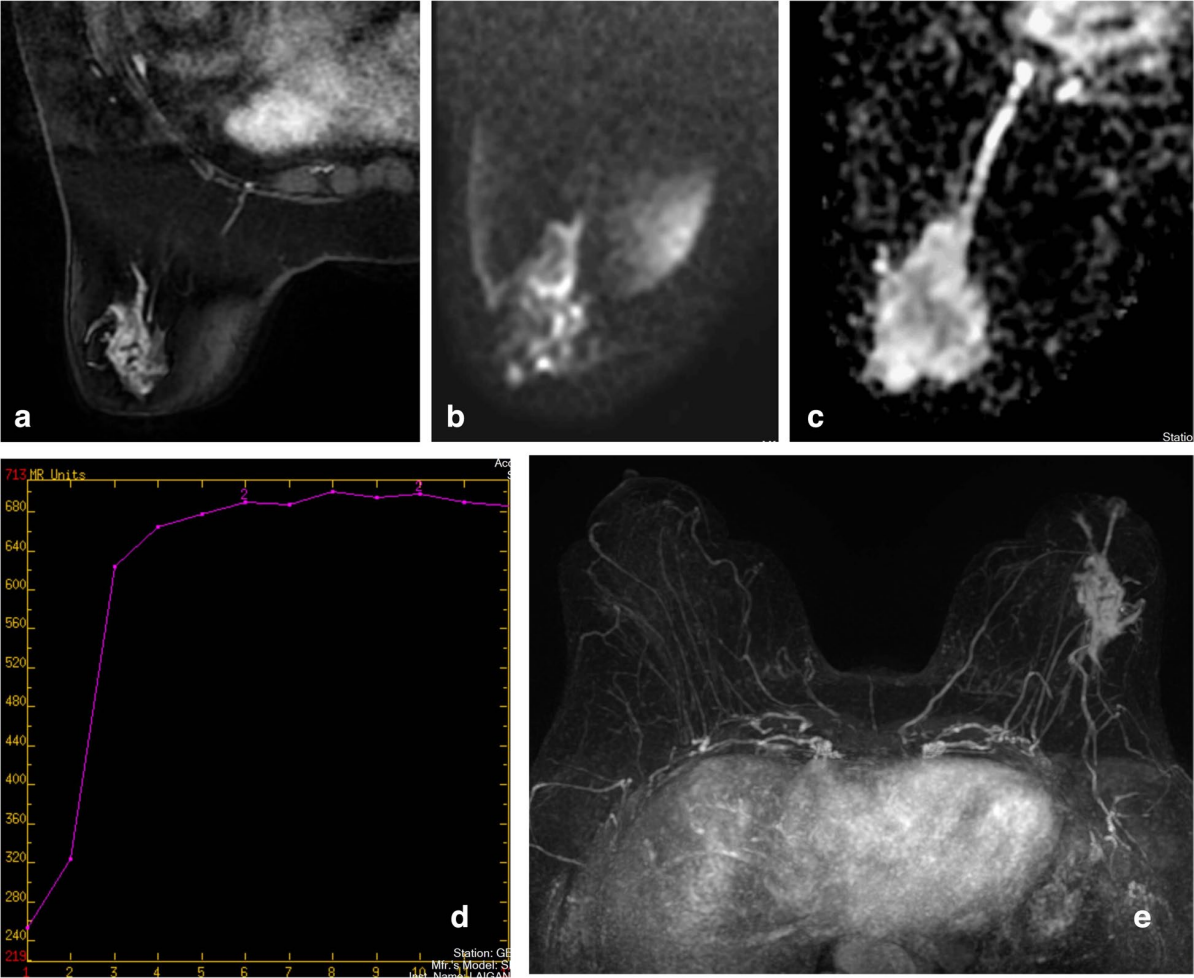
**a** A lesion in a 58-year-old female with segmental distribution and heterogeneous enhancement. **b** and **c** The ADC value was 1.58 × 10^–3^ mm^2^/s, **d** The imaging revealed an typeII TIC curve. **e** The imaging revealed feeding vessels. The multiparametric score was 4the lesion was determined as BI-RADS-MRI type 4c. The pathological finding was hyperplasia of the left breast with intraductal carcinoma in situ

The distribution, T2WI signal, internal enhancement pattern, early enhancement rate, enhancement curve, ADC value, and vascular sign of the lesions were included in the logistic multivariate analysis. It was found that the distribution (odds ratio (OR) = 8.70), internal enhancement pattern (OR = 6.29), ADC value (OR = 4.56), and vascular sign (OR = 2.84) of the lesions were closely related to the benignity and malignancy of the lesions, and the differences had statistical significance (*P* < 0.05). The above signs were included in the multimodal scoring: 4 point for a segmental lesion, and 0 points for lesions with other distributions;3 point for clustered enhancement, and 0 points for other enhancement types; 2point for ADC with a value less than 1.40, otherwise 0 points; and 1 points for the presence of vascular signs, otherwise 0 points. ROC analysis revealed that its optimal diagnostic cut-off value was 5. ROC analysis revealed that the diagnostic specificity and sensitivity were 87.01% and 82.22%, respectively. All lesions were subjected to multimodal scoring, and the details are shown in Table 2 and Fig. 2. Lesions with 1–6 points were considered BI-RADS category 4 lesions, and the positive predictive values of subtypes 4a, 4b, and 4c lesions were 15.79%, 31.25%, and 77.78%, respectively. For details, see Table 3 and Figs. 3 and 4.

**Table 2:** Multiparametric scoring criteria for non-mass-like enhancement lesions

	Sign	Score
Distribution	Segmental distribution (including the distribution along the duct)	4
	Nonsegmental distribution	0
Internal enhancement pattern	Clustered enhancement	3
	Nonclustered enhancement	0
ADC value	> 1.40 × 10^–3^ mm^2^/s	0
	≤ 1.40 × 10^–3^ mm^2^/s	2
Vascular sign	Yes	1
	None	0

**Table 3:** Results of multiparametric scoring of non-mass-like enhancement lesions

	Category 3 (0 point)	Category 4A (1-2point)	Category 4B (3-4point)	Category 4C (5-6point)	Category 5 (≥ 7point)
Benign	10	16	11	4	3
Malignant	1	3	5	14	32
Total	11	19	16	18	35
Positive predictive value		15.79%	31.25%	77.78%

## Discussion

Our results of multivariate logistic regression analysis showed that the distribution, internal enhancement pattern, ADC value, and positive vascular signs of the lesions were independent predictors of malignant lesions. Therefore, we assigned 4,3,2,1 points, respectively. A multiparametric prediction model was established by assigning points to various factors. ROC analysis revealed that the diagnostic specificity and sensitivity were 87.01% and 82.22%, respectively. Moreover, we preliminarily subclassified the category 4 lesions and defined 1 and 2point as 4a, 3 and 4 points as 4b, and 5and 6 points as 4c; the predictive values of positive malignant lesions were 15.79%, 31.25%, and 77.78%, respectively, which roughly met the provisions of BI-RADS and demonstrated that our prediction model had good diagnostic efficacy. The positive predictive value of 4a lesions was 15.79%, slightly higher than the BI-RADS, which may be due to less lesions.

Of the 77 malignant lesions in this group, 57 had segmental distribution, suggesting that segmental distribution has a high diagnostic value for malignant lesions, which is consistent with previous studies [10–12]. The reason for this result is related to the growth of the tumor along the duct. Of the 77cases of malignant NMLE, 46 cases showed clumped enhancement (46/77), suggesting that clumped enhancement is a more reliable sign of malignant lesions, which is consistent with previous reports. However, some studies have shown that the internal enhancement pattern of malignant lesions is mainly clustered ring enhancement and clustered enhancement [12–15], while only 9 cases in our group of patients showed clustered ring enhancement, and 7 of them had benign lesions, which may be because our determination of clustered ring enhancement was different from these other studies. In this study, we considered lesions with clustered ring enhancement in the first phase of enhancement as having clustered ring enhancement. Among the 9 patients with clustered ring enhancement in this group, 6 had inflammation (3 had abscess formation and 2 had ductal dilatation), and the causes of their clustered ring enhancement were abscess or periductal inflammatory cell infiltration, leading to increased vascular permeability. During the early stage, abscess or periductal enhancement was visible, which may be the reason why our results were different from those reported previously. Some scholars determine clustered ring enhancement in the late arterial and late enhancement phases and believe that its pathological basis is due to a decrease in the degree of tumor enhancement with the outflow of contrast agent after early enhancement, whereas the breast ductal wall and surrounding stroma show delayed enhancement, so clustered ring enhancement occurs [14, 16, 17]. Some scholars also suggest that clustered ring enhancement should be judged within 2 min of enhancement because the enhancement of the gland after delay will affect the judgment [18]. Therefore, there is disagreement on the determination of clustered ring enhancement, and it is necessary to collect additional cases to investigate and study.

As a functional imaging method, DWI can reflect the changes in water molecule diffusion capacity at the molecular level in different tissues and in the same tissues under different states. In malignant tumors, the ADC values are usually smaller than those in benign lesions because of the dense distribution of cells, large nuclei and scant plasma, and limited diffusion of water molecules. In this group of lesions, we also obtained the same results: the ADC value of benign lesions was significantly higher than that of malignant lesions (1.37 ± 0.41 vs. 1.14 ± 0.25).In our study, the cut-off point of ADC value is 1.40 × 10^−3^mm^2^/s, higher than some other study [8, 9], because we only analyzed NMLE. The weight of ADC value of this group of lesions is significantly lower than the distribution and internal enhancement pattern. Considering that it is related to the intermingling of tumors and normal tissues in NMLE lesions, the measurement data is difficult to be as accurate as that of MLE lesions. The ADC value of lesions in this group was obtained by selecting multiple points for the measurement and taking the average value in order to avoid bias in the data as much as possible.

By scoring the number of blood vessels adjacent to the lesion using the MRI maximum intensity projection technique, we found that abnormally increased and thickened blood vessels or feeding vessels were present within and next to the lesion. This positive adjacent vascular sign indicates the possibility of malignant lesions. Among the 77 malignant lesions of this group, 54 showed this sign, whereas the incidence of this sign in benign lesions was low, with the majority of these lesions being abscesses. We believe that the vascular sign reflects the blood supply of the breast tumor at the macroscopic level; its presence is not affected by the breast glandular tissue, and it can directly influence the blood supply of malignant tumor. Therefore, the increased and thickened blood vessels around the lesion may have more advantages than the TIC curve and early enhancement rate in identifying the benignity and malignancy of lesions.

In Logistic Analysis, the TIC was excluded. Because most of the benign and malignant lesions of NMLE in this group showed a type II curve (80/122), and most of malignant lesions were type II.(56/71),however, more than half benign (24/45) lesions have type II curve.. And the Chi square analysis showed that there was no significant difference in early enhancement rate between benign and malignant NMLE lesions. The reason may be that the NMLE lesions often contain some normal glandular tissues and fat components, which will lead to the partial volume effect, so the measurement results are not accurate to a certain extent.

Conventional T1WI and T2WI can help determine whether there is cystic degeneration, necrosis, or hemorrhage in the lesion and evaluate whether there is infiltration of skin, ligaments, pectoralis major muscle, and other structures as well as whether there is peritumoral edema. In this group of patients, we found that the signal of NMLE lesions on T2WI was variable without significant specificity and was mostly slightly hyperintense. We also agree with the view of Gong et al. [19] that it is not advisable to position and qualitatively diagnose the lesion with the signal characteristics of a conventional noncontrast MRI scan alone.

However, this study still has the following limitations: 1. The sample size of this study was small, so statistical bias was unavoidable. 2. The DCE-MRI parameters( such as K^trans^, K_ep_, Ve, and TTP) and mammography, were not analyzed. 3. This study is a retrospective study, which has certain subjectivity in the judgment of NMLE. Thus, the consistency of diagnoses by different physicians should be further analyzed in the future.

## Conclusions

The multiparametric differential diagnosis model established in this study can improve the accuracy of NMLE lesion diagnosis and provide physicians with a reliable method that can be rapidly mastered for NMLE classification and subclassification.

## Data Availability

The datasets used and/or analyzed during the current study are available from the corresponding author on reasonable request.

## Abbreviations

NMLE: Non-mass-like enhancement
OR: Odds ratio
MRI: Magnetic resonance imaging
BI-RADS: Breast Imaging Reporting and Database System
DWI: Diffusion-weighted imaging
EPI: Echo-planar imaging
TR: Repetition time
TE: Echo time
NEX: Number of excitations
T2WI: T2-weighted imaging
FSE: Fast spin-echo
Gd-DTPA: Gadolinium-diethylenetriamine pentaacetic acid
TIC: Time-intensity curve
ADC: Apparent diffusion coefficient
ROC: Receiver operating characteristic
